# Proteomic and immunoproteomic characterization of a DIVA subunit vaccine against *Actinobacillus pleuropneumoniae*

**DOI:** 10.1186/1477-5956-9-23

**Published:** 2011-04-20

**Authors:** Falk FR Buettner, Sarah A Konze, Alexander Maas, Gerald F Gerlach

**Affiliations:** 1 Department of Infectious Diseases, Institute for Microbiology, University of Veterinary Medicine Hannover, Bischofsholer Damm 15, 30173 Hannover, Germany; 2 Department of Cellular Chemistry, Hannover Medical School, Carl-Neuberg-Strasse 1, 30625 Hannover, Germany; 3 vaccinova GmbH, Abschnede 64, 27472 Cuxhaven, Germany; 4 IVD GmbH, Heisterbergallee 12, 30453 Hannover, Germany

## Abstract

**Background:**

Protection of pigs by vaccination against *Actinobacillus pleuropneumoniae*, the causative agent of porcine pleuropneumonia, is hampered by the presence of 15 different serotypes. A DIVA subunit vaccine comprised of detergent-released proteins from *A. pleuropneumoniae *serotypes 1, 2 and 5 has been developed and shown to protect pigs from clinical symptoms upon homologous and heterologous challenge. This vaccine has not been characterized in-depth so far. Thus we performed i) mass spectrometry in order to identify the exact protein content of the vaccine and ii) cross-serotype 2-D immunoblotting in order to discover cross-reactive antigens. By these approaches we expected to gain results enabling us to argue about the reasons for the efficacy of the analyzed vaccine.

**Results:**

We identified 75 different proteins in the vaccine. Using the PSORTb algorithm these proteins were classified according to their cellular localization. Highly enriched proteins are outer membrane-associated lipoproteins like OmlA and TbpB, integral outer membrane proteins like FrpB, TbpA, OmpA1, OmpA2, HgbA and OmpP2, and secreted Apx toxins. The subunit vaccine also contained large amounts of the ApxIVA toxin so far thought to be expressed only during infection. Applying two-dimensional difference gel electrophoresis (2-D DIGE) we showed different isoforms and variations in expression levels of several proteins among the strains used for vaccine production. For detection of cross-reactive antigens we used detergent released proteins of serotype 7. Sera of pigs vaccinated with the detergent-released proteins of serotypes 1, 2, and 5 detected seven different proteins of serotype 7, and convalescent sera of pigs surviving experimental infection with serotype 7 reacted with 13 different proteins of the detergent-released proteins of *A. pleuropneumoniae *serotypes 1, 2, and 5.

**Conclusions:**

A detergent extraction-based subunit vaccine of *A. pleuropneumoniae *was characterized by mass spectrometry. It contained a large variety of immunogenic and virulence associated proteins, among them the ApxIVA toxin. The identification of differences in expression as well as isoform variation between the serotypes implied the importance of combining proteins of different serotypes for vaccine generation. This finding was supported by immunoblotting showing the induction of cross-reactive antibodies against several surface associated proteins in immunized animals.

## Background

*Actinobacillus pleuropneumoniae *is a gram-negative rod-shaped bacterial pathogen causing porcine pleuropneumonia, a highly contagious disease for pigs which is often fatal and leads to severe economic losses in the swine industry worldwide [1]. Transmission occurs by aerosols or close contact with infected pigs [2] and it can cause mild to severe clinical signs depending on serotype, infectious dose, and immune status of the animal. Acute infection is characterized by hemorrhagic, fibrinous pneumonia often leading to rapid death of affected animals. Pigs surviving the infection can become chronically infected asymptomatic carriers of *A. pleuropneumoniae*. Carrier animals, in which the pathogen is able to persist for months in sequestered lung tissue and in tonsils, can transmit the disease to healthy animals and are the major source for new outbreaks [3,4].

*A. pleuropneumoniae *strains are categorized into two biotypes based on their dependency on exogenous NADH. Biotype 1 strains are NADH auxotrophic whereas strains belonging to biotype 2 are capable of synthesizing NADH autonomously. Fifteen different serotypes have been described on the basis of capsular antigens [5]. All serotypes are obligate pathogens, but differ in virulence [6] and regional distribution. Serotypes 1, 5 and 7 are predominant in North America, serotype 2 is most common to Europe [7] and serotypes 1, 3, 4, 5 and 7 are typically isolated in China [8]. This diversity has hampered vaccination against porcine pleuropneumonia and there is no fully efficacious vaccine offering complete protection against all serotypes to date. Vaccination against bacterial pathogens has gained increasing relevance due to growing problems with antibiotic resistance and rising consumer demands concerning food safety [9].

Commercial vaccines against *A. pleuropneumoniae *infection mostly consist of whole cell bacterins [9]. Although reducing mortality, they commonly do not prevent infection or development of the carrier state. Additionally, protection is limited to the serotype(s) used in the vaccine preparation, and differentiation between infected and vaccinated animals is not possible [4,9-11]. The reproducible production of bacterin vaccines essentially depends on standard production routines for procedures such as culture and inactivation of the bacteria. However, the molecular composition of bacterin vaccines is generally not known. Thereby quality control and comparison between different production lots is limited.

To overcome these limitations subunit vaccines have been developed for different bacteria containing a subset of bacterial antigens [9]. Generally, these antigens are immunogenic, expressed *in vivo*, and - to confer cross-serotype protection - are conserved among different strains. To discover new potential vaccine components for *A. pleuropneumoniae *the outer membrane proteome has been resolved [12] and immunogenic outer membrane as well as secreted proteins have been identified [13,14]. Also, gene expression *in vivo *has been analyzed, and protein expression studies under conditions mimicking the *in vivo *situation (e.g. iron restriction, addition of BALF, anaerobic conditions) have been reported [15-19].

An *A. pleuropneumoniae *subunit vaccine was developed from cultures grown under iron restriction by mild detergent extraction of proteins without lysing the bacteria [20]. This subunit vaccine was postulated to be enriched in outer membrane lipoproteins as suggested by the presence of transferrin binding lipoprotein B (TbpB) and the outer membrane lipoprotein A (OmlA) whereas periplasmic, cytoplasmic and membrane proteins were absent [20]. Vaccination of pigs with this vaccine derived from *A. pleuropneumoniae apxIIA*-mutants of serotypes 1, 2 and 5 showed good protective efficacy upon homologous challenge with serotype 2 and cross-protection against serotype 9 and facilitated the differentiation between infected and vaccinated animals [21].

In this study we set out to fully characterize the subunit vaccine by addressing the following questions: i) What is the exact protein composition of the vaccine? ii) Which proteins might be responsible for cross-serotype protectivity? Thus we analyzed the serotype-specific vaccine components by ultra performance liquid chromatography-coupled tandem mass spectrometry (UPLC MS/MS) and two-dimensional difference gel electrophoresis (2-D DIGE). We additionally tested the efficacy of the subunit vaccine to induce antibodies detecting homologous proteins of a different serotype and used *A. pleuropneumoniae *serotype 7 for that purpose.

## Results

### Proteomic analysis of vaccine components

For characterization of the subunit vaccine we used a strategy designated "whole vaccine-proteome approach". A single preparation of detergent released proteins (designation: "detergent-wash") of each of the *A. pleuropneumoniae *serotypes contained in the vaccine was separated by SDS-PAGE (Additional file 1, Figure S1) and analyzed by UPLC-coupled Q-TOF MS/MS.

In each analysis several hundred masses were automatically subjected to fragmentation and determination of false discovery rate (FDR) revealed values of above 5% erroneously identified proteins when including "single-peptide-identifications". Only one protein was identified from the decoy database searches by two peptide matches. Thus, for the whole vaccine-proteome approach we defined a threshold of at least two peptide matches for sufficient protein identification. This approach led to the identification of 47, 43, and 36 different proteins for the "detergent-wash" of *A. pleuropneumoniae *serotypes 1, 2 and 5, respectively. In total 64 different proteins were identified by at least two peptide matches in the "detergent wash" from serotypes 1, 2, and 5. Thirty-two of these proteins were present in the preparations of each of the three strains (Additional file 2, Table S1). Fourteen proteins were identified by at least 10 peptide matches in one of the serotypes (Table 1). These proteins were designated as "abundant protein fraction" of the subunit vaccine.

**Table 1:** List of the "abundant protein fraction" identified in the *A. pleuropneumoniae *"detergent wash" preparations upon 1-D PAGE.

Protein description ^a)^	Protein ^a)^	Accession # ^a)^	Peptide matches ^b)^				PSORTb localization ^c)^	PSORTb score ^c)^	Characteristics ^d)^
			**Subunit vaccine**						
			**Ser 1**	**Ser 2**	**Ser 5**	**Ser 7**			

RTX toxin protein	ApxIA	Q548V0	**24**	**(1) ^e)^**	**29**	(1) ^e)^	Extracellular	10	Immunogenic [40]
RTX toxin protein	ApxIIIA	P55130	**(2) ^e)^**	**26**	**(1) ^e)^**	(1) ^e)^	Extracellular	10	Immunogenic [13]
Iron regulated outer membrane protein B	FrpB	B3H0B8	**18**	**15**	**21**	17	OuterMembrane	10	Immunogenic [13]
Outer membrane lipoprotein A	OmlA	B3GYZ9	**19**	**14**	**10**	19	OuterMembrane	9.92	Immunogenic [42]
Transferrin binding protein	TbpB	B3GYQ1	**14**	**14**	**19**	17	OuterMembrane	9.49	Immunogenic [41]
RTX toxin protein	ApxIVA	B3H1M8	**10**	**16**	**15**	0	Extracellular	10	Immunogenic [30]; expressed *in vivo *[34]; upregulated *in vivo *[36]
Transferrin binding protein 1 Tbp1	TbpA	B3GYQ0	**7**	**4**	**16**	11	OuterMembrane	10	Immunogenic [41]; expressed *in vivo *[34]
Elongation factor Tu	TufB1, TufB2	B3GYJ3	**16**	**14**	**16**	15	Cytoplasmic	9.97	Immunogenic [13]; expressed *in vivo *[34,35]
Outer membrane protein P5	OmpA1	B3H2D9	**9**	**9**	**14**	10	OuterMembrane	10	Immunogenic [13]; expressed *in vivo *[34]; *in vivo *survival [37]; antigenic and *in vivo *expressed [14]
Elongation factor G	FusA	B3H2G7	**4**	**12**	**12**	6	Cytoplasmic	9.97	
Outer membrane protein P5 OMP P5	OmpA2	B3GZA8	**10**	**11**	**10**	7	OuterMembrane	10	Immunogenic [13]; expressed *in vivo *[34]; *in vivo *survival [37]; antigenic and *in vivo *expressed [14]
Hemoglobin binding protein A	HgbA	B3H1S8	**10**	**6**	**0**	9	OuterMembrane	10	Expressed *in vivo *[34]
Outer membrane protein P2	OmpP2	B3H172	**10**	**9**	**10**	8	OuterMembrane	10	*in vivo *survival [38]
Iron Chelated ABC transporter periplasmic binding protein	YfeA	B3H0B4	**6**	**9**	**10**	10	Periplasmic	10	Immunogenic [13]

Protein identifications in the individual "detergent washes" of the *A. pleuropneumoniae *serotypes 1, 2 and 5 used for generation of the subunit vaccine ("whole vaccine-proteome approach", highlighted in bold) and serotype 7.

^a) ^In order to have a consistent nomenclature for proteins identified from *A. pleuropneumoniae *serotypes 1, 2, 5 or 7 in the columns "protein description", "protein" and "accession number", the annotation of the respective homologue of *A. pleuropneumoniae *serotype 7 was adopted from the uniprot knowledgebase http://www.uniprot.org/. For the Apx toxins ApxIA and ApxIIIA, which are not present in *A. pleuropneumoniae *serotype 7, the common nomenclature was used.

^b) ^Peptide matches in the serotype specific vaccine components identified by UPLC-coupled Q-TOF MS/MS

^c) ^Subcellular prediction using the PSORTb v.3.0 tool at http://www.psort.org/psortb/[53]. A probability based score from 0 (low probability) to 10 (high probability) is given.

^d) ^The identified proteins were searched in the literature for homologues identified in *A. pleuropneumoniae *as immunogenic, virulence-associated or relevant *in vivo*.

^e) ^These peptides from Apx toxins that are not present in the respective preparations because they are not encoded by the respective serotype were merely identified because they are identical or highly homologous to peptides from other Apx toxins that are present in the respective preparations.

Analysis of the cellular location using PSORTb version 3.0 http://www.psort.org/psortb/ revealed that eight of 14 proteins of the "abundant protein fraction" are outer membrane proteins (FrpB, OmlA TbpB, TbpA, OmpA1, OmpA2, HgbA and OmpP2). High numbers of peptide matches were also obtained for the extracellular Apx toxins IA, IIIA and IVA contained in the "detergent-wash". Additionally, two cytosolic proteins (TufB2 and FusA) as well as a periplasmic protein (YfeA) were identified with more than 10 peptide matches (Table 1).

### Comparison of serotype-specific vaccine components by 2-D DIGE

**Figure 1:**
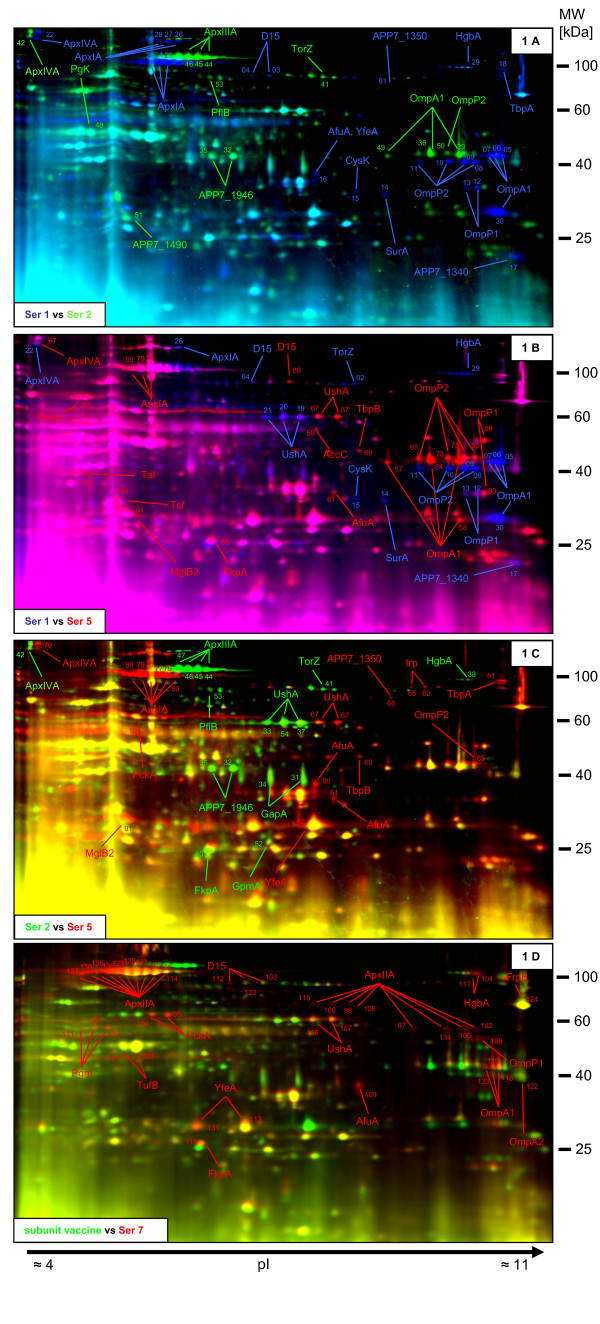
**2-D DIGE of "detergent-wash" proteins from *A. pleuropneumoniae *serotypes 1, 2 and 5 (subunit vaccine) and serotype 7**. For analysis of the subunit vaccine serotype 1, 2, and 5 were labelled with Cy2 (shown in blue), Cy3 (shown in green) and Cy5 (shown in red), respectively, and subsequently pooled. For visualization of differences between the serotypes used for vaccine generation, we compared couples of serotypes 1 and 2 (A), serotypes 1 and 5 (B), and serotypes 2 and 5 (C). For comparison of the subunit vaccine to serotype 7, the subunit vaccine was labelled with Cy3 (shown in green) and the "detergent wash" of serotype 7 was labelled with Cy5 (shown in red, D). Spots with intensities considerably above that in one or the other serotype were analyzed by mass spectrometry from preparative gels of the respective serotype (Additional file 4, Figure S2). The annotation of the identified protein is given in the same colour as the labelling of the respective serotype. The numbers on each spot, that has been identified, are consecutive and allow the finding of the respective spot on preparative gels (Additional file 4, Figure S2).

The vaccine components from *A. pleuropneumoniae *serotypes 1, 2 and 5 were labelled with different fluorescent dyes and separated by 2-dimensional gel electrophoresis (2-DE) on the same gel (Figure 1A-C). As we only used a single protein preparation and did not intend to perform an exact quantification but rather describe the content of the subunit vaccine, only spots showing obvious differences in spot intensity between the three strains were used for spot selection. Quantitative differences between spots of the three different serotypes used for vaccine generation are provided additionally (Additional file 3, Table S2). Spots were picked from respective preparative gels (Additional file 4, Figure S2), trypsinized and analyzed by either UPLC-coupled ESI Q-TOF MS/MS or MALDI-TOF MS (Additional file 3, Table S2).

Q-TOF MS/MS analyses of single spots from 2-D gels resulted in almost no false positive discoveries as generally fewer spectra were subjected to database searches than in the "whole vaccine-proteome approach". Thus, we also included "single-peptide-identifications" as results but confirmed these further according to the procedure for MALDI-TOF MS results not having a significant score (as described in Methods).

For *A. pleuropneumoniae *serotypes 1, 2 and 5 mass spectrometry was successful for 30, 26 and 39 spots, respectively, and led to the identification of 16, 16 and 22 different proteins. In total 36 different proteins were identified from gels of the three *A. pleuropneumoniae *serotypes (Additional file 3, Table S2). Eleven of these 36 proteins were not identified by the "whole vaccine-proteome approach" using a threshold of at least two peptide identifications (Additional file 2, Table S1). A combination of the "whole vaccine-proteome approach" and the gel-based approach resulted in the identification of a total of 75 different proteins present in the subunit vaccine.

Series of spots that appear as horizontal strings were generally identified as belonging to the same protein. Especially the Apx toxins were represented by several spots in each serotype. Adjacent spots from different serotypes having only a slight shift in pI or mass were also often identified as homologous proteins. For example UshA of *A. pleuropneumoniae *serotype 5 is represented by two additional spots adjacent to those of *A. pleuropneumoniae *serotypes 1 and 2. Also, the outer membrane proteins OmpA1, OmpP1 and OmpP2 differ in mass and pI among the different *A. pleuropneumoniae *serotypes (Figure 1A-C).

### Serotype-specific protein expression

We analyzed the combined data from the "whole vaccine-proteome approach" (Table 1, Additional file 2, Table S1) and 2-D DIGE (Additional file 3, Table S2) to compare protein expression between the different *A. pleuropneumoniae *serotypes. As controls the Apx protein profiles were analyzed. As expected, the ApxIA toxin was identified from gels of *A. pleuropneumoniae *serotype 1 and 5 as well as by the "whole vaccine-proteome approach" but not from *A. pleuropneumoniae *serotype 2 by both methods. The ApxIIIA toxin was only present on *A. pleuropneumoniae *serotype 2 gels and identified by the "whole vaccine-proteome approach" from this serotype only. ApxIVA was identified from 2-DE gels of *A. pleuropneumoniae *serotypes 1, 2 and 5 as well as by the "whole vaccine-proteome approach" from these serotypes.

The hemoglobin binding protein HgbA was found on 2-DE gels from *A. pleuropneumoniae *serotypes 1 and 2 as well as by the "whole vaccine-proteome approach" with 10 and six peptides, respectively, but HgbA was not found for *A. pleuropneumoniae *serotype 5 by either approach. UshA was represented by more prominent spots on gels of *A. pleuropneumoniae *serotypes 1 and 2 compared to serotype 5. This protein was identified by the "whole vaccine-proteome approach" from *A. pleuropneumoniae *serotypes 1 and 2 with five and four peptides, respectively, but only by one peptide from serotype 5. On the other hand, the D-galactose-binding protein MglB2 was represented by a prominent spot on the *A. pleuropneumoniae *serotype 5 gel and identified by four peptides from serotype 5 exclusively. Expression of the transferrin binding protein 1 (TbpA) was identified by 2-D DIGE as increased in *A. pleuropneumoniae *serotypes 1 and 5 compared to serotype 2; this finding was supported by the result of the "whole vaccine-proteome approach".

The "whole vaccine-proteome approach" revealed the identification of the autotransporter adhesins AtaB and AtaA by five peptides and two peptides, respectively, only from serotype 2. Additionally, homologues to the uncharacterized protein APP7_1946 and the hybrid peroxyredoxin protein APP7_1490 were represented by more prominent spots on *A. pleuropneumoniae *serotype 2 gels. UPLC-coupled tandem mass spectrometry supported this observation as five peptides were identified for APP7_1946 and APP7_1490 in serotype 2 but only one peptide was identified for each protein from *A. pleuropneumoniae *serotype 1 and none from serotype 5 preparations.

### Identification of cross-reactive antigens by immunoproteomics

In order to test the efficacy of the subunit vaccine to induce cross-serotype reactive antibodies, we used "detergent-wash" proteins from serotype 7 strain AP76. For that purpose the "detergent-wash" of serotype 7 was characterized in advance and was analyzed as described for the vaccine containing serotypes 1, 2, and 5 by 1-D electrophoresis (Additional file 1, Figure S1) followed by UPLC-coupled ESI Q-TOF MS/MS, which led to the identification of 33 different proteins (Table 1, Additional file 2, Table S1). Most of the proteins that had been identified for serotype 7 were also present in the subunit vaccine. For the majority of the identified proteins the number of peptide matches was in the same range as found for the vaccine strains. As we used a serotype 7 wild type strain we could identify the ApxIIA protein which was absent from the vaccine strains that were *apxIIA *deletion mutants. Additionally the uncharacterized protein APP7_1217 was only found in serotype 7. In contrast to serotypes 1, 2, and 5 no ApxIVA protein was identified in the serotype 7 "detergent-wash" (Table 1, Additional file 2, Table S1).

Applying 2-D DIGE we compared the "detergent-wash" of *A. pleuropneumoniae *serotype 7 and the subunit vaccine (Figure 1D). ApxIIA of serotype 7 was identified on the gel as a series of horizontal spots. Some serotype 7 proteins had similar masses but different isoelectric points as compared to proteins of the subunit vaccine. PckA and UshA and HgbA had more alkaline isoelectric points whereas YfeA was shifted to the acidic side (Figure 1D).

**Figure 2:**
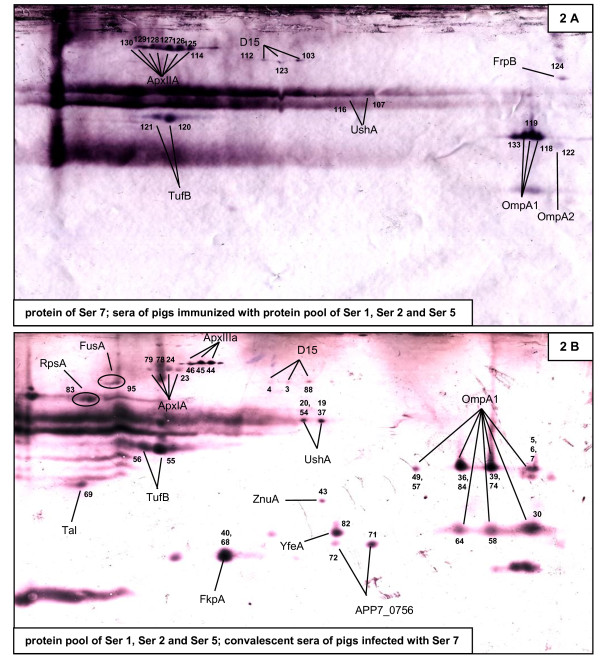
**Immunoblot analysis for the identification of cross-reactive proteins**. "Detergent-wash" proteins were separated by 2-D and subsequently Western blotted. "Detergent-wash" proteins from serotype 7 were probed using immune sera of pigs immunized with the subunit vaccine (A) and the subunit vaccine was probed with convalescent sera from pigs upon experimental infection with serotype 7 (B). Immunogenic spots were assigned to spots on preparative Coomassie stained gels (Additional file 4, Figure S2) and identified by mass spectrometry. The numbers on each spot that has been identified allow the finding of the respective spot on preparative gels (Additional file 4, Figure S2).

For the identification of cross-reactive antibodies induced by vaccination with the subunit vaccine we performed immunoblotting of serotype 7 "detergent-wash" proteins and used sera of pigs immunized with the subunit vaccine (Figure 2A). These sera detected seven different proteins from serotype 7 (ApxIIA, D15, UshA, FrpB, TufB, OmpA1, and OmpA2).

To compare the immunological efficacy of vaccination with the subunit vaccine to a natural infection we subsequently probed subunit vaccine proteins with convalescent sera of pigs experimentally infected with *A. pleuropneumoniae *serotype 7 (Figure 2B) and detected 13 different proteins. The proteins D15, UshA, TufB, OmpA1 were found in both cases. The convalescent sera further detected RpsA, FusA, ApxIA, ApxIIIA, Tal, FkpA, ZnuA, YfeA and APP7_0756. As controls we used different protein preparations of serotype 7 and did immunoblotting with sera of pigs that were neither infected with *A. pleuropneumoniae *nor immunized with the subunit vaccine. These sera detected only OmpA, OmpP2 and ApxIIA very weakly and considerably less than upon infection or vaccination (data not shown).

We tested the conservation of the detected cross-reactive proteins and compared serotype 7 strain AP76 to serotype 1 strain 4047, serotype 2 strain 4226, and serotype 5b strain L20 for which genomic sequences are available on NCBI by doing "BLAST align two sequences" analyses. The identities on the amino acid level were above 98% for all proteins except for the Apx toxins which are not present in all serotypes and the elongation factors RpsA and TufB which were truncated or discontinuous in serotype 2.

## Discussion

Treatment of *A. pleuropneumoniae *grown under iron restriction with the mild detergent sodium deoxycholate has been introduced as a suitable method for enrichment of antigenic outer membrane-associated lipoproteins [20]. A preparation of proteins obtained by this method derived from *apxIIA *deletion mutants of *A. pleuropneumoniae *serotypes 1, 2 and 5 was used as a DIVA subunit vaccine and protected pigs from clinical signs upon homologous challenge with *A. pleuropneumoniae *serotype 2 and heterologous challenge with serotype 9 [21]. However, the protein composition of this subunit vaccine has not been determined so far and, therefore, was addressed in this study using state-of-the-art proteomic technologies.

A combination of different bioinformatic tools predicted 93 outer membrane proteins for *A. pleuropneumoniae *serotype 5b [12] and searching the serotype 5b genome with the SLEP pipeline (http://www.caspur.it/slep[22]), which optimally combines several prediction tools, revealed even more outer membrane or secreted proteins. Our analyses led to the identification of 75 different proteins of which 15 proteins are membrane proteins and four are secreted proteins as predicted by the PSORTb algorithm. Thus several predicted outer membrane and secreted proteins were not identified by our approach, which is likely due to their low concentration or even absence in the vaccine preparation.

As a semi-quantitative measure we postulate a correlation between identified peptide matches and protein abundance. Of 64 proteins identified in total by UPLC Q-TOF MS/MS, 14 were identified by 10 or more peptide matches. These proteins were therefore designated as the "abundant protein fraction". Among these three Apx toxins and eight outer membrane proteins were found. This approach showed that the detergent extraction not only enriched outer membrane-associated lipoproteins like OmlA and TbpB [20], but also integral outer membrane proteins like FrpB, TbpA, OmpA1, OmpA2, HgbA and OmpP2. Only three of the highly enriched proteins are cytosolic (TufB and FusA) or periplasmic (YfeA). Their percentage is higher in the fraction of low abundant vaccine proteins and might be caused by lysed cells as well as by the release of membrane vesicles containing these proteins [23].

A combined interpretation of the "whole vaccine-proteome approach" and the 2-D DIGE analysis revealed different amounts for several proteins in the "detergent-wash" preparations of the three serotypes, indicating serotype dependent differences in gene expression. Additionally, the 2-DIGE analysis showed the presence of various isoforms for several proteins among and between the different strains that vary slightly in mass or isoelectric point. For proteins represented by different isoforms, a high ratio-value often means that only the respective isoform is abundant in one or the other strain. However, this does not necessarily refer to the total amount of the respective protein, which holds true e.g. for the outer membrane protein OmpP2. This protein was identified by comparable peptide numbers between the different serotypes on the one hand, but also appeared in several isoforms with high ratio-values in each strain on the other hand.

With an exception of ApxIIA, all secreted Apx toxins (including ApxIVA) produced by the *A. pleuropneumoniae *strains used for vaccine generation were detected, and they represent the most abundant protein fraction of the analyzed subunit vaccine. ApxIIA was not identified as *apxIIA *gene deletion mutants of *A. pleuropneumoniae *serotypes 1, 2 and 5 had been used for vaccine generation in order to generate a DIVA vaccine [21].

The Apx toxins are important for virulence [24,25] and subunit vaccines based on Apx toxins have been developed, eg. Porcillis APP™ (Intervet, [26]). Therefore, the high content of ApxIA, ApxIIIA and ApxIVA in the analyzed subunit vaccine is presumably one important factor accounting for the protective efficacy of the "detergent-wash" vaccine.

ApxIA and ApxIIIA were identified on 2-DE gels as series of up to 10 spots with different isoelectric points, which is likely caused by charged posttranslational modifications (PTMs). Different isoforms of other proteins were also present within and between strains. As it cannot be excluded that these isoforms vary with respect to their protective efficacy for vaccination purposes, *A. pleuropneumoniae*-derived proteins might be superior in comparison to recombinant proteins.

ApxIVA has been considered as a DIVA antigen as it is immunogenic and encoded by most *A. pleuropneumoniae *isolates [27-29]. It was shown for serotype 7 strain AP76 that the *apxIV*A gene is interrupted by an insertion element preventing *in vivo *expression of *apxIVA *[29]. This is in accordance with our data showing no ApxIVA protein in the "detergent-wash" of AP76. Expression of the *apxIVA *gene has been considered for long as strictly induced *in vivo *[30] and was shown recently to be upregulated by bronchoalveolar fluid [19,31]. We show for the first time the translation of the *apxIVA *gene and identified abundant amounts of the respective protein in the "detergent-washes" of *A. pleuropneumoniae *serotypes 1, 2, and 5. This presence of ApxIVA in the analyzed subunit vaccine is likely to contribute to immune protection of vaccinated pigs [32].

As we have grown *A. pleuropneumoniae *for vaccine generation under iron restrictive conditions, one might argue that these conditions induce *apxIVA *expression. However, in contrast to many other proteins that were highly enriched in the subunit vaccine, iron restrictive induction of *apxIVA *has not been observed while analyzing the transcriptional profile of *A. pleuropneumoniae *serotype 5b during iron restriction grown in BHI-medium [17]. We therefore speculate that the ApxIVA expression might be due to using customized growth conditions (iron-restriction in combination with supplemented PPLO-medium, see in Methods). Other reports describing the lack of *apxIVA *expression in culture either used BHI-medium [13] or PPLO-medium supplemented with horse serum [30] for growth of *A. pleuropneumoniae*.

The "detergent-wash" vaccine contains several proteins encoded by genes only expressed or upregulated *in vivo *as identified by IVET [33], SCOTS [34,35] or transcriptional profiling [36]. Proteins relevant for *in vivo *survival of *A. pleuropneumoniae *that were identified by STM [37,38] are also present in the vaccine. Six of the abundant proteins were shown previously to be induced *in vivo *during acute infection (ApxIVA, TbpA, TufB, OmpA1, OmpA2, and HgbA, [34]). Further, TufB, AasP, DnaK, GapA and RplC had been identified as expressed in chronically infected lung by *A. pleuropneumoniae *[35]. Furthermore, the proteins OmpP2, ZnuA and APP7_2020 are considered as being virulence-associated, since the respective STM mutants were attenuated [38]. A recent study on transcriptional profiling of *A. pleuropneumoniae *during the acute phase of infection detected four of the proteins identified in the "detergent-wash", namely TolB, ApxIVA, APP7_0979, and Irp [36]. APP7_0979 is a conserved lipoprotein [12,39] and was among the highest *in vivo *induced proteins [36].

The analyzed subunit vaccine was highly enriched in immunogenic proteins. A recent immunoproteomic analysis of secreted and outer membrane proteins from *A. pleuropneumoniae *serotype 3 revealed 30 immunogenic proteins [13] of which 18 were also identified in the analyzed subunit vaccine. Six of these proteins (ApxIIIA, FrpB, TufB, OmpA1, OmpA2 and YfeA) were among the 14 most abundant proteins; an additional five proteins among the 14 most abundant proteins were identified as immunogenic elsewhere including the ApxIA [40] and ApxIVA [30] toxin, as well as the iron repressible transferring binding proteins TbpA and TbpB [41] and the outer membrane lipoprotein OmlA [42].

As the immunoproteome of *A. pleuropneumoniae *has been extensively studied [13] we set out to identify cross-reactive antigens. Immunization of pigs with the presented subunit vaccine comprised of serotypes 1, 2, and 5 led to formation of antibodies against seven different serotype 7 proteins of which six (TufB, ApxIIA, D15, FrpB, OmpA1, OpmA2) were already known [13] except for UshA, which is a periplasmic protein catalyzing the degradation of external UDP-glycose to uridine, glycose-1-phosphate and inorganic phosphate [43].

Convalescent sera of pigs obtained upon experimental infection with serotype 7 detected 13 immunogenic proteins of the subunit vaccine of which seven were already described as immunogenic (TufB, D15, OmpA1, ApxIIIA, FkpA, ZnuA [13], and ApxIA [40]). The proteins RpsA, Tal, APP7_0756, FusA, YfeA, and UshA are cross-reactive immunogenic antigens that have not been described for *A. pleuropneumoniae *before. YfeA is the periplasmic binding protein of an ABC transport system for iron and manganese acquisition and deletion of this ABC system renders *Yersinia pestis *avirulent [44]. In the porcine pathogen *Haemophilus parasuis*, which is related to *A. pleuropneumoniae*, YfeA has been recently identified as an cross-reactive immunogenic protein [45]. Based on BLAST analysis, APP7_0756 is also the periplasmic component of an ABC transport system needed for iron acquisition.

In total, infection with a live *A. pleuropneumoniae *led to formation of more cross-reactive antibodies than immunization using the described subunit vaccine. Thus several proteins were only detected by convalescent sera (RpsA, FusA, ApxIA, ApxIIIA, Tal, FkpA, ZnuA, YfeA and APP7_0756), although they are present in the subunit vaccine. Live bacteria seem to induce an even stronger immune response than the subunit vaccine, which is in accordance with the findings that pigs surviving natural or experimental infection are at least partially protected from clinical symptoms upon infection with another serotype [46,47].

The analyzed subunit vaccine was comprised of detergent extracted proteins from three different serotypes of *A. pleuropneumoniae *in order to increase the number of serotype-specific antigens. Additionally, the likelihood that a serotype specific antigen from one or the other serotype is cross-reactive against another serotype was increased. BLAST analysis showed that the identified immunogenic proteins were highly conserved between the different *A. pleuropneumoniae *serotypes explaining the observed cross-reactivity. Several of the identified proteins are ideally suited as vaccine components against *A. pleuropneumoniae *based on their conservation [14,26].

The different Apx toxins of *A. pleuropneumoniae *are cross-reactive although sharing low homology. Immune sera of pigs vaccinated with the subunit vaccine, detected the ApxIIA toxin from serotype 7, although ApxIIA was not present in the subunit vaccine. On the other hand convalescent sera of pigs infected with serotype 7, which exclusively expresses the *apxIIA *gene, detected the ApxIA and ApxIIIA proteins of the vaccine. The Apx toxins share as a common structural feature a tandem array of a nine amino acid repeat [48] which might cause their cross-reactivity.

## Conclusions

We characterized the protein components of a non-recombinant DIVA subunit vaccine against *A. pleuropneumoniae *prepared by "detergent-wash" and we showed that secreted and outer membrane proteins were highly enriched with many of these proteins being proven as immunogenic, transcribed *in vivo *or required for survival in the host. The ApxIVA protein was for the first time identified from *in vitro *grown *A. pleuropneumoniae *as part of the subunit vaccine. The subunit vaccine led to formation of cross-reactive antibodies and contained several antigens that have not been described as immunogenic before. These results rationalize the cross-serotype protective efficacy of the "detergent-wash"-based subunit vaccine observed previously.

## Methods

### Bacterial strains and growth conditions

*A. pleuropneumoniae *strains were cultured at 37°C in a shaking incubator at 180 rpm in PPLO medium (Difco, Augsburg, Germany) supplemented with NAD (10 μg/ml; Merck, Darmstadt, Germany), L-cysteine hydrochloride (260 μg/ml; Sigma, Deisenhofen, Germany), L-cystine dihydrochloride (10 μg/ml; Sigma), dextrose (1 mg/ml) and Tween^® ^80 (0.1%).

### "Detergent-wash" extraction of surface-associated proteins from iron restricted cultures for application as a subunit vaccine

For preparation of surface-associated proteins, *apxIIA *deletion mutants of *A. pleuropneumoniae *serotype 1 (ATCC 27088), 2 (DM322/5), 5 (K1711/84) [21] and the wild type of serotype 7 (AP76, [49]) were incubated in 150 ml of supplemented PPLO medium to an OD_600 _of 0.2. Then diethylentriamine-pentaacetic acid calcium trisodium salt hydrate (Na_3_CaDTPA, Fluka Chemika and BioChemika, Germany, permitted as a drug component for livestock) was added to a final concentration of 150 μM in order to induce iron restrictive conditions. Bacteria were further grown to an OD_600 _of 0.6 - 0.7 followed by addition of 10 mM Tris-HCl (pH 8.0), 50 mM NaCl and 0.05% [w/v] of the mild detergent sodium deoxycholate (Sigma, permitted as a drug component for livestock). After incubation for 30 min at 37°C by shaking at 180 rpm, cultures were centrifuged for 15 min at 8,000 × g to remove cells. The cell-free culture supernatant (CFS), containing surface-associated proteins, was sterile-filtrated (0.22 μm pore size filter, Millipore, Billerica, USA) and stored at -20°C until further use [20,21]. Proteins from supernatants were precipitated with trichloracetic acid (TCA; 20% [w/v] final concentration) overnight and harvested by centrifugation (15,000 × g, 30 min). Protein pellets were washed with acetone and solubilized for 1-D PAGE in 50 mM Tris-HCl (pH 7.2), 150 mM NaCl or for 2-D PAGE in a buffer containing 30 mM Tris-HCl pH 8.5, 7 M urea [Roth, Karlsruhe, Germany], 2 M thiourea [Sigma], 4% [wt/vol] CHAPS [Roth]. Insoluble material was removed by centrifugation at 16,000 × g for 5 min in a benchtop centrifuge. Protein concentration was determined using the Pierce 660 nm Protein Assay (Thermo, Pierce, Rockford, USA).

### "Whole vaccine-proteome approach" by 1-D PAGE UPLC-coupled Q-TOF MS/MS

40 μg protein of each serotype was loaded on a 10.8% polyacrylamide gel. After gel electrophoresis each lane was cut into four slices and subjected to tryptic in-gel digestion as described below. Each digest was then analyzed by nanoUPLC-coupled Q-TOF tandem mass spectrometry as described below with the following amendment: As samples were still complex, we analyzed each digest up to three times. Peptides that have been fragmented once were excluded from fragmentation in the following mass spectrometric analyses, in order to enable fragmentation of low intensity peptides. As the second or third run generally led to only a few new peptide identifications the sample was considered as comprehensively analyzed. This analysis was designated the "whole vaccine-proteome approach".

### 2-D gel electrophoresis and 2-D DIGE

For 2-D DIGE 100 μg protein of *A. pleuropneumoniae *serotypes 1, 2 and 5 were labeled with Cy2, Cy3 and Cy5, (GE Healthcare, Freiburg, Germany, according to the manufacturer's instructions) respectively, pooled and subsequently separated on the same 2-D gel. On preparative gels 300 μg protein of each of the vaccine-specific serotypes was separated individually and subsequently stained with Coomassie G-250 [50].

2-D gel electrophoresis, scanning of DIGE gels and data analysis were performed as described previously [16]. Briefly, Immobiline^® ^DryStrips (GE Healthcare) of 24 cm length with a pI gradient from 3 to 11 were used. Samples were loaded into anodal sample cups and subsequently focused using an Ettan™ IPGphor™ (GE Healthcare). For second dimension the IPG strips were placed on the top of 12.5% polyacrylamide gels that had been precast with low-fluorescent glass plates (GE Healthcare) using an Ettan™ DALTsix gel caster (GE Healthcare). The second-dimension SDS-PAGE was carried out using the Ettan™ Daltsix Electrophoresis System (GE Healthcare). Gels with fluorescently labeled proteins were scanned on a Typhoon Trio™ scanner (GE Healthcare). Relative spot intensities between the different serotypes were compared using the DIA tool of the DeCyder™-Software (GE Healthcare).

### In-gel digestion

Spots of interest were excised from preparative gels, trypsinized and peptides were recovered according to standard procedures [51]. Briefly, the gel pieces were dehydrated with acetonitrile (ACN, Merck), rehydrated with NH_4_HCO_3 _buffer (100 mM) containing DTT (10 mM; Roth), and then treated with iodoacetamide (100 mM; Sigma) in NH_4_HCO_3 _(100 mM). Dehydration and rehydration were repeated, and then dehydrated gel pieces were rehydrated with buffer (50 mM NH_4_HCO_3_) containing trypsin (20 ng/μl, sequencing grade; Promega, Mannheim, Germany) and incubated for 12 - 16 h at 37°C. Peptides were extracted using NH_4_HCO_3 _(50 mM) followed by extraction buffer containing ACN (50% [vol/vol]; Merck) and formic acid (5% [vol/vol]; Merck). The solution containing the extracted peptides was evaporated in a vacuum centrifuge.

### Protein identification by UPLC-coupled Q-TOF MS/MS or MALDI-TOF MS

For ESI Q-TOF tandem mass spectrometry peptides were dissolved in water, and reverse phase chromatography using acetonitril as an eluent was performed on a Waters Aquity nanoUPLC (Milford, USA) device equipped with an analytical column (Waters, BEH130C18, 100 μm × 100 mm, 1.7 μm particle size) coupled online to an ESI Q-TOF (Q-TOF Ultima, Waters). Peptide spectra were recorded in positive reflection mode and peptides were automatically subjected to fragmentation (MS/MS). Protein identification was performed using the program ProteinLynx™ Global Server (Version 2.1, Waters) by searching against a *A. pleuropneumoniae *genome database (actinobacillus+pleuropneumoniae+taxonomy_715.fasta) downloaded at http://www.uniprot.org/ from the Protein Knowledgebase (UniProtKB) on 27.12.2009 containing 6552 entries (2026 entries of serotype 3 strain JL03, 2004 entries of serotype 5b strain L20, and 2117 entries of serotype 7 strain AP76). This database contained the complete genomes of the serotypes 3, 5, and 7 that were available at the date of analysis and was applied for identification of proteins obtained from serotype 1, 2, 5 and 7.

Alternatively, MALDI-TOF MS was carried out on a VoyagerDE Pro workstation (Applied Biosystems, USA). Peptides were dissolved in ACN (50% [vol/vol], Merck), formic acid (0.1% [vol/vol] Merck), mixed with an equal volume of matrix (α-cyano-4-hydroxy-cinnamic acid [Bruker Daltonics, Billerica, USA], 5 mg/ml in 50% acetonitrile with 0.1% trifluoroacetic acid) and then spotted on the target plate. Peptide spectra were acquired in positive reflection mode, averaging about 1000 laser shots per MALDI-TOF spectrum. Mass spectra were calibrated using the calibration mixtures CalMix1 and CalMix2 (Applied Biosystems). Peptide mass lists were generated from peptide spectra using the Applied Biosystems Data Explorer^® ^V 4.8 software. The peptide mass lists were used to perform the peptide mass fingerprint algorithm on the Mascot web site http://www.matrixscience.com by searching against the NCBI non-redundant database or a respective sub-database. Typically, the search algorithm was set to allow carbamidomethylation on cysteine residues, oxidation on methionine residues, and a maximum of 1 missed cleavage. The peptide mass tolerance was up to 40 ppm. Proteins were considered as identified when the probability-based score was above the significance threshold (p ≤ 0.05). For some proteins we could not obtain a sufficiently high score. If further criteria were fulfilled we included these proteins into the list of identifications. Thus, we compared the calculated molecular mass and isoelectric point values from the identified proteins with the observed values on the 2-D gel. Additionally, if a protein with a non-significant score was part of a horizontal series of spots with adjacent identical proteins we included this identification.

### Quality evaluation of proteomics results

For quality evaluation of proteomics results we determined the false discovery rate (FDR), applying the online version of the Decoy Database Buildner software tool (http://www.medizinisches-proteom-center.de, [52]). As a target database we used the *A. pleuropneumoniae *database actinobacillus+pleuropneumoniae+taxonomy_715.fasta. A decoy database was generated by reversing the amino acid sequences in the target database. The spectra were then searched against this decoy database. The FDR is calculated as the number of different proteins identified from the decoy database search (false positives, FP) divided by the sum of FP and the number of different proteins identified from the target database search (true positives, TP; FDR = FP/(FP + TP)).

### Immunoblotting

CyDye™ (GE Healthcare) labeled proteins were separated by 2-D gel electrophoresis and transferred to nitrocellulose membranes (Protran BA85 0.45 μm, Schleicher and Schuell, Dassel, Germany) using the MultiphorTM II system (GE Healthcare). The blotting membrane was incubated with porcine sera. Convalescent sera were obtained from pigs 21 days post experimental infection with *A. pleuropneumoniae *serotype 7 strain AP76. Immune-sera of pigs vaccinated with the subunit vaccine were obtained six weeks after initial and three weeks after booster immunization. Sera were diluted 1:100 in PBST (8 g NaCl, 0.2 g KH_2_PO_4_, 1.44 g Na_2_HPO_4_, 0.2 g KCl, 0.5 ml Tween 20, ad 1000 ml). As second antibody an alkaline phosphatase-conjugated goat anti swine IgG antibody (Jackson ImmunoResearch Laboratories, Dianova, Hamburg, Germany) was used in a 1:5,000 dilution. BCIP (5-bromo-4-chloro-3-indol phosphate) and NBT (nitro blue tetrazolium) were used as substrates for visualization of immunogenic proteins. Immunogenic proteins on Western blots were assigned to corresponding spots on preparative Coomassie-stained gels, which were run in parallel with the same protein preparation, allowing mass spectrometric identification of the respective proteins.

## Competing interests

The authors declare that they have no competing interests.

## Authors' contributions

FFRB performed DIGE experiments, mass spectrometry and immunoassays, and drafted the manuscript. SAK contributed to protein analytics. AM carried out the preparation of the subunit vaccine. GFG and FFRB conceived of the study and participated in its design and coordination. All authors read and approved the final manuscript.

## Supplementary Material

Additional file 1 **Figure S1: 1-D PAGE separation of "detergent-wash" proteins**. "Detergent-wash" proteins from serotypes 1, 2 and 5 (subunit vaccine) and from serotype 7 were separated on a polyacrylamide gel. Each lane was divided into 4 pieces (A-D) and proteins were in-gel digested with trypsin, extracted from the gel piece and then analyzed by UPLC-coupled Q-TOF tandem mass spectrometry. Click here for file

Additional file 2 **Table S1: List of all proteins identified in the *A. pleuropneumoniae *"detergent wash" preparations upon 1-D PAGE**. Click here for file

Additional file 3 **Table S2: List of proteins identified from 2-D gels**. Click here for file

Additional file 4 **Figure S2: Preparative 2-D gels of "detergent-wash" proteins**. "Detergent-wash" proteins of serotypes 1, 2, 5 and 7 were separated on individual 2-D gels. Proteins were stained with colloidal Coomassie blue and proteins of interest were picked from these gels, trypsinized and analyzed by mass spectrometry. The numbers on each spot, that has been identified, are consecutive and allow the finding of the respective spot in Figure 1 and 2. Click here for file
