# Supplementary figures and images for: Proteomic and immunoproteomic characterization of a DIVA subunit vaccine against Actinobacillus pleuropneumoniae
Source: Proteome Sci. 2011 Apr 20;9:23. doi: 10.1186/1477-5956-9-23 (PMC3107771; doi:10.1186/1477-5956-9-23)

## Slide 1
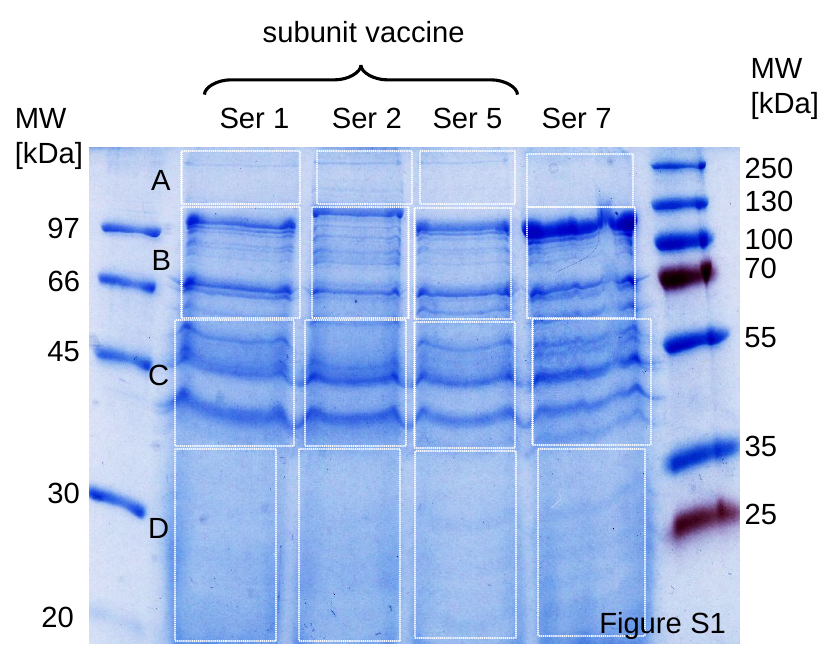

subunit vaccine
MW [kDa]
MW [kDa]
Ser 1
Ser 2
Ser 5
Ser 7
250
A
130
97
100
B
70
66
55
45
C
35
30
25
D
20
Figure S1

Supplement: Additional file 1 — Figure S1: 1-D PAGE separation of "detergent-wash" proteins. "Detergent-wash" proteins from serotypes 1, 2 and 5 (subunit vaccine) and from serotype 7 were separated on a polyacrylamide gel. Each lane was divided into 4 pieces (A-D) and proteins were in-gel digested with trypsin, extracted from the gel piece and then analyzed by UPLC-coupled Q-TOF tandem mass spectrometry. [file 1477-5956-9-23-S1.PPT]
