# Supplementary material for: Proteomic and immunoproteomic characterization of a DIVA subunit vaccine against Actinobacillus pleuropneumoniae
Source: Proteome Sci. 2011 Apr 20;9:23. doi: 10.1186/1477-5956-9-23 (PMC3107771; doi:10.1186/1477-5956-9-23)
Supplement: Additional file 2 — Table S1: List of all proteins identified in the A. pleuropneumoniae "detergent wash" preparations upon 1-D PAGE. [file 1477-5956-9-23-S2.DOC]

**Table S1. List of all proteins identified in the *A. pleuropneumoniae* “detergent wash” preparations upon 1-D PAGE.**

| **Protein description ^a)^** | **Protein ^a)^** | **Accession # ^a)^** | **Peptide matches ^b)^** | | | | **PSORTb localization ^c)^** | **PSORTb score ^c)^** | **Characteristics ^d)^** |
| --- | --- | --- | --- | --- | --- | --- | --- | --- | --- |
|  |  |  | **Subunit vaccine** | | |  |  |  |  |
|  |  |  | **Ser 1** | **Ser 2** | **Ser 5** | **Ser 7** |  |  |  |
| RTX toxin protein | ApxIA | Q548V0 | **24** | **(1) ^e)^** | **29** | (1) ^e)^ | Extracellular | 10 | Immunogenic [40] |
| RTX toxin protein | ApxIIIA | P55130 | **(2) ^e)^** | **26** | **(1) ^e)^** | (1) ^e)^ | Extracellular | 10 | Immunogenic [14] |
| Iron regulated outer membrane protein B | FrpB | B3H0B8 | **18** | **15** | **21** | 17 | OuterMembrane | 10 | Immunogenic [14] |
| Outer membrane lipoprotein A | OmlA | B3GYZ9 | **19** | **14** | **10** | 19 | OuterMembrane | 9.92 | Immunogenic [42] |
| Transferrin binding protein | TbpB | B3GYQ1 | **14** | **14** | **19** | 17 | OuterMembrane | 9.49 | Immunogenic [41] |
| RTX toxin protein | ApxIVA | B3H1M8 | **10** | **16** | **15** | 0 | Extracellular | 10 | Immunogenic [30]; expressed *in vivo* [34]; upregulated *in vivo* [36] |
| Transferrin binding protein 1 Tbp1 | TbpA | B3GYQ0 | **7** | **4** | **16** | 11 | OuterMembrane | 10 | Immunogenic [41]; expressed *in vivo* [34] |
| Elongation factor Tu | TufB1, TufB2 | B3GYJ3 | **16** | **14** | **16** | 15 | Cytoplasmic | 9.97 | Immunogenic [14]; expressed *in vivo* [34,35] |
| Outer membrane protein P5 | OmpA1 | B3H2D9 | **9** | **9** | **14** | 10 | OuterMembrane | 10 | Immunogenic [14]; expressed *in vivo* [34]; *in vivo* survival [37]; antigenic and *in vivo* expressed [15] |
| Elongation factor G | FusA | B3H2G7 | **4** | **12** | **12** | 6 | Cytoplasmic | 9.97 |  |
| Outer membrane protein P5 OMP P5 | OmpA2 | B3GZA8 | **10** | **11** | **10** | 7 | OuterMembrane | 10 | Immunogenic [14]; expressed *in vivo* [34]; *in vivo* survival [37]; antigenic and *in vivo* expressed [15] |
| Hemoglobin binding protein A | HgbA | B3H1S8 | **10** | **6** | **0** | 9 | OuterMembrane | 10 | Expressed *in vivo* [34] |
| Outer membrane protein P2 | OmpP2 | B3H172 | **10** | **9** | **10** | 8 | OuterMembrane | 10 | *in vivo* survival [38] |
| Iron Chelated ABC transporter periplasmic binding protein | YfeA | B3H0B4 | **6** | **9** | **10** | 10 | Periplasmic | 10 | Immunogenic [14] |
| Putative uncharacterized protein | APP7_0979 | B3GXR6 | **9** | **0** | **8** | 11 | Unknown | 2 | Upregulated *in vivo* [36] |
| Outer membrane receptor protein | APP7_1966 | B3H2U8 | **1** | **0** | **8** | 4 | OuterMembrane | 9.49 |  |
| Phosphoglycerate kinase | Pgk | B3H222 | **7** | **7** | **6** | 5 | Cytoplasmic | 9.97 |  |
| Adenylate kinase | Adk | B3H271 | **6** | **2** | **3** | 3 | Cytoplasmic | 9.97 |  |
| Lipoprotein 5 domain containing protein | APP7_2045 | B3H327 | **3** | **0** | **6** | 0 | Extracellular | 9.65 |  |
| Peptidyl prolyl cis trans isomerase | FkpA | B3H2N7 | **6** | **1** | **1** | 1 | Periplasmic | 9.76 | Immunogenic [14]; *in vivo* survival [37] |
| Ribosome recycling factor | Frr | B3GXB4 | **6** | **0** | **0** | 2 | Cytoplasmic | 9.97 |  |
| Putative outer membrane protein precursor | OmpP1 | B3GXS8 | **4** | **1** | **6** | 3 | OuterMembrane | 9.95 |  |
| High affinity zinc uptake system protein | ZnuA | B3H2B9 | **5** | **3** | **6** | 7 | Periplasmic | 10 | Immunogenic [14]; *in vivo* survival [38] |
| ABC transport system periplasmic protein | APP7_0756 | B3GXE9 | **5** | **4** | **5** | 6 | CytoplasmicMembrane | 9.97 | Immunogenic [14] |
| Hybrid peroxiredoxin HyPrx5 | APP7_1490 | B3H2B7 | **1** | **5** | **0** | 0 | Unknown | 2 |  |
| Putative uncharacterized protein | APP7_1946 | B3GZB2 | **1** | **5** | **0** | 0 | Unknown | 2.5 |  |
| Autotransporter adhesin | AtaB | B3GZU4 | **0** | **5** | **0** | 0 | Unknown | 5.87 | Expressed *in vivo* [34] |
| Enolase OS | Eno | B3GY00 | **0** | **5** | **2** | 0 | Cytoplasmic | 9.97 |  |
| Adenylosuccinate synthetase | PurA | B3H1V8 | **0** | **5** | **0** | 0 | Cytoplasmic | 9.97 |  |
| 50S ribosomal protein L1 | RplA | B3GYU5 | **5** | **5** | **4** | 1 | Cytoplasmic | 9.26 |  |
| UshA | UshA | B3GXM3 | **5** | **4** | **1** | 4 | Periplasmic | 9.76 |  |
| Autotransporter serine protease | AasP | B3H0M6 | **4** | **1** | **2** | 7 | OuterMembrane | 9.83 | Expressed *in vivo* [35] |
| Putative uncharacterized protein | APP7_1340 | B3H262 | **4** | **2** | **2** | 3 | Unknown | 2.5 | Immunogenic [14] |
| Outer membrane antigenic lipoprotein B | APP7_2020 | B3H302 | **3** | **4** | **1** | 1 | OuterMembrane | 8.86 | Immunogenic [14]; *in vivo* survival [38] |
| Autotransporter adhesin | AtaA | B3GZU3 | **0** | **4** | **0** | 0 | Unknown | 5.87 |  |
| Putative periplasmic binding protein CbiK | CbiK | B3H2M1 | **4** | **3** | **3** | 2 | Unknown | 2 | Immunogenic [14] |
| Chaperone protein dnaK | DnaK | B3H2X7 | **2** | **4** | **3** | 0 | Cytoplasmic | 9.97 | Expressed *in vivo* [34,35]; *in vivo* survival [37] |
| 3 oxoacyl acyl carrier protein synthase 1 | FabB | B3H1T7 | **4** | **2** | **0** | 1 | Cytoplasmic | 9.97 |  |
| Fructose bisphosphate aldolase | Fba | B3H221 | **3** | **3** | **4** | 2 | Cytoplasmic | 9.97 |  |
| Glyceraldehyde 3 phosphate dehydrogenase | GapA | B3H0Z9 | **4** | **3** | **1** | 2 | Cytoplasmic | 9.97 | Immunogenic [14]; expressed *in vivo* [35] |
| D galactose binding periplasmic protein | MglB2 | B3H2E0 | **0** | **0** | **4** | 0 | Periplasmic | 9.76 | Immunogenic [14] |
| 50S ribosomal protein L3 | RplC | B3GZ11 | **2** | **2** | **4** | 1 | Cytoplasmic | 9.26 | Expressed *in vivo* [35] |
| Translocation protein TolB | TolB | B3H0E7 | **3** | **4** | **3** | 2 | Periplasmic | 9.44 | Upregulated *in vivo* [36] |
| Trimethylamine N oxide reductase | TorZ | B3H1B3 | **1** | **4** | **0** | 1 | Periplasmic | 10 |  |
| Putative uncharacterized protein | APP7_1901 | B3GZ67 | **3** | **3** | **3** | 3 | OuterMembrane | 10 |  |
| Thiol disulfide interchange protein DsbC | DsbC | B3GX35 | **1** | **0** | **3** | 0 | Periplasmic | 9.76 |  |
| Phosphoenolpyruvate carboxykinase ATP | PckA | B3H1D9 | **2** | **3** | **2** | 2 | Cytoplasmic | 9.97 |  |
| Superoxide dismutase | SodA | B3H093 | **1** | **3** | **0** | 1 | Periplasmic | 9.44 |  |
| Acetate kinase | AckA | B3H169 | **0** | **2** | **2** | 5 | Cytoplasmic | 9.97 |  |
| Putative uncharacterized protein | APP7_0395 | B3H0N5 | **2** | **0** | **0** | 0 | Unknown | 2.5 |  |
| Putative uncharacterized protein | APP7_1413 | B3GYE1 | **2** | **1** | **0** | 1 | OuterMembrane | 9.52 | Antigenic and *in vivo* expressed [15] |
| Peptidyl prolyl cis trans isomerase | APP7_1766 | B3GYT2 | **2** | **1** | **1** | 0 | Cytoplasmic | 9.26 |  |
| 2 3 cyclic nucleotide 2 phosphodiesterase | CpdB | B3H170 | **2** | **1** | **0** | 0 | Periplasmic | 9.44 |  |
| 2 3 4 5 tetrahydropyridine 2 6 dicarboxylate N succinyltransferase | DapD | B3GZB7 | **0** | **2** | **0** | 0 | Cytoplasmic | 9.97 |  |
| Malate dehydrogenase | Mdh | B3H269 | **1** | **2** | **0** | 0 | Unknown | 4.99 | Immunogenic [14] |
| Fe S biogenesis protein nfuA | NfuA | B3GZZ1 | **2** | **0** | **0** | 0 | Cytoplasmic | 9.97 |  |
| Transcription antitermination protein | NusG | B3GYU3 | **2** | **0** | **0** | 0 | Cytoplasmic | 9.97 | Induced in vivo [33] |
| Phosphoglucomutase phosphomannomutase | Pgm | B3H119 | **2** | **0** | **0** | 0 | Cytoplasmic | 9.26 |  |
| Lipoprotein Plp4 | PlpD | B3GX37 | **2** | **1** | **0** | 0 | OuterMembrane | 9.92 |  |
| Ribose 5 phosphate isomerase A | RpiA | B3H285 | **2** | **0** | **0** | 1 | Unknown | 2 |  |
| 50S ribosomal protein L6 | RplF | B3GZ26 | **2** | **2** | **2** | 0 | Cytoplasmic | 9.26 |  |
| Peptidyl prolyl cis trans isomerase | SlyD | B3H288 | **2** | **0** | **1** | 2 | Cytoplasmic | 9.97 |  |
| Transketolase 2 | TktA | B3GXX6 | **0** | **2** | **0** | 0 | Cytoplasmic | 9.97 |  |
| Elongation factor Ts | Tsf | B3GXB1 | **0** | **0** | **2** | 0 | Cytoplasmic | 9.97 |  |
| RTX toxin protein | ApxIIA | B3GXU5 | **0** | **(2) ^e)^** | **(3) ^e)^** | 53 | Extracellular | 10 |  |
| Putative uncharacterized protein | APP7_1217 | B3GY46 | **0** | **0** | **0** | 11 | OuterMembrane | 8.86 |  |
| ABC type Fe3 transport system periplasmic component | AfuA | B3GYH9 | **0** | **0** | **0** | 2 | Periplasmic | 9.76 |  |
| **Total protein identifications (> 2 peptides)** |  |  | **47** | **43** | **36** | **33** |  |  |  |
| **Sum of different proteins in serotypes 1, 2, and 5 (subunit vaccine)** |  |  | **64** | | |  |  |  |  |
| **False discoveries having at least 2 peptide matches using a reverse decoy database** |  |  | **0** | **0** | **1** | **0** |  |  |  |

Protein identifications in the individual „detergent washes“ of the *A. pleuropneumoniae* serotypes 1, 2 and 5 used for generation of the subunit vaccine (“whole vaccine-proteome approach”, highlighted in bold) and serotype 7.

^a)^ In order to have a consistent nomenclature for proteins identified from *A. pleuropneumoniae* serotypes 1, 2, 5 or 7 in the columns “protein description”, “protein” and “accession number”, the annotation of the respective homologue of *A. pleuropneumoniae* serotype 7 was adopted from the uniprot knowledgebase (http://www.uniprot.org/). For the Apx toxins ApxIA and ApxIIIA, which are not present in *A. pleuropneumoniae* serotype 7, the common nomenclature was used.

^b)^ Peptide matches in the serotype specific vaccine components identified by UPLC-coupled Q-TOF MS/MS

^c)^ Subcellular prediction using the PSORTb v.3.0 tool at http://www.psort.org/psortb/ [53]. A probability based score from 0 (low probability) to 10 (high probability) is given.

^d)^ The identified proteins were searched in the literature for homologues identified in *A. pleuropneumoniae* as immunogenic, virulence-associated or relevant *in vivo*.

^e)^ These peptides from Apx toxins that are not present in the respective preparations because they are not encoded by the respective serotype were merely identified because they are identical or highly homologous to peptides from other Apx toxins that are present in the respective preparations.
