# Supplementary material for: Proteomic and immunoproteomic characterization of a DIVA subunit vaccine against Actinobacillus pleuropneumoniae
Source: Proteome Sci. 2011 Apr 20;9:23. doi: 10.1186/1477-5956-9-23 (PMC3107771; doi:10.1186/1477-5956-9-23)
Supplement: Additional file 3 — Table S2: List of proteins identified from 2-D gels. [file 1477-5956-9-23-S3.DOC]

**Table S2: List of proteins identified from 2-D gels.**

| **Sero-type** | **Spot #** | **Annotation in serotype 7 str. AP76 (NC_010939)** | | | **mW** | **pI** | **Relative spot intensities** | |
| --- | --- | --- | --- | --- | --- | --- | --- | --- |
|  |  | **Protein description ^a) b)^** | **Protein ^a)^** | **UniProtKB accession # ^a)^** |  |  | **Ser 1 / Ser 2** | **Ser 1 / Ser 5** |
| **Sero-type**  **1** | 1 | Predicted TonB dependent Ligand Gated channel | APP7_1350 | B3H272 | 77203 | 8.5 | **2.4** |  |
|  | 2 | Trimethylamine N oxide reductase | TorZ | B3H1B3 | 91202 | 7.3 |  | **6.6** |
|  | 3 | **Protective surface antigen D15** | D15 | B3H0S5 | 89132 | 6.6 | **2.1** |  |
|  | 4 | **Protective surface antigen D15** | D15 | B3H0S5 | 89132 | 6.6 | **5.0** | **2.4** |
|  | 5 | **Outer membrane protein P5** | OmpA1 | B3H2D9 | 39588 | 9.3 | **9.4** | **42.8** |
|  | 6 | **Outer membrane protein P5** | OmpA1 | B3H2D9 | 39588 | 9.3 | **13.9** | **60.9** |
|  | 7 | **Outer membrane protein P5** | OmpA1 | B3H2D9 | 39588 | 9.3 | **6.6** | **14.4** |
|  | 8 | Outer membrane protein P2 | OmpP2 | B3H172 | 39497 | 9.1 | **5.2** | **10.6** |
|  | 9 | Outer membrane protein P2 | OmpP2 | B3H172 | 39497 | 9.1 | **6.3** | **16.1** |
|  | 10 | Outer membrane protein P2 | OmpP2 | B3H172 | 39497 | 9.1 | **8.5** | **11.7** |
|  | 11 | Outer membrane protein P2 | OmpP2 | B3H172 | 39497 | 9.1 | **3.2** | **5.9** |
|  | 12 | Putative outer membrane protein | OmpP1 | B3GXS8 | 52907 | 9.2 | **3.8** | **2.1** |
|  | 13 | Putative outer membrane protein | OmpP1 | B3GXS8 | 52907 | 9.2 | **2.9** | **2.3** |
|  | 14 | Peptidyl prolyl cis trans isomerase | SurA | B3H0R4 | 34562 | 8.4 | **7.7** | **7.0** |
|  | 15 | Cysteine synthase | CysK | B3H090 | 33722 | 8.3 | **3.8** | **6.3** |
|  | 16 | ABC type Fe3 transport system periplasmic component | AfuA | B3GYH9 | 37793 | 8.4 | **3.7** |  |
|  | 16 | Iron Chelated ABC transporter periplasmic binding protein | YfeA | B3H0B4 | 32893 | 7.5 | **3.7** |  |
|  | 17 | Putative uncharacterized protein | APP7_1340 | B3H262 | 23548 | 9.7 | **4.2** | **3.3** |
|  | 18 | Transferrin binding protein 1 Tbp1 | TbpA | B3GYQ0 | 106999 | 9.2 | **3.3** |  |
|  | 19 | **5'-nucleotidase/2',3'-cyclic phosphodiesterase and related esterases** | UshA | B3GXM3 | 60857 | 7.1 |  | **5.2** |
|  | 20 | **5'-nucleotidase/2',3'-cyclic phosphodiesterase and related esterases** | UshA | B3GXM3 | 60857 | 7.1 |  | **6.9** |
|  | 21 | 5'-nucleotidase/2',3'-cyclic phosphodiesterase and related esterases | UshA | B3GXM3 | 60857 | 7.1 |  | **9.4** |
|  | 22 | RTX-IV toxin determinant A | ApxIVA | B3H1M8 | 149447 | 4.5 | **6.1** |  |
|  | 23 | **RTX I toxin protein determinant A** | ApxIA | Q548V0 | 109967 | 5.5 | **27.0** |  |
|  | 24 | **RTX I toxin protein determinant A** | ApxIA | Q548V0 | 109967 | 5.5 | **39.5** |  |
|  | 25 | RTX I toxin protein determinant A | ApxIA | Q548V0 | 109967 | 5.5 | **2.2** |  |
|  | 26 | RTX I toxin protein determinant A | ApxIA | Q548V0 | 109967 | 5.5 | **2.3** | **6.4** |
|  | 27 | RTX I toxin protein determinant A | ApxIA | Q548V0 | 109967 | 5.5 | **6.0** |  |
|  | 28 | RTX I toxin protein determinant A | ApxIA | Q548V0 | 109967 | 5.5 | **4.6** |  |
|  | 29 | Hemoglobin binding protein A | HgbA | B3H1S8 | 107545 | 9.0 | **2.3** | **19.6** |
|  | 30 | **Outer membrane protein P5** | OmpA1 | B3H2D9 | 39588 | 9.3 | **5.8** | **8.3** |
|  |  |  |  |  |  |  |  | |
|  |  |  |  |  |  |  | **Ser 2 / Ser 1** | **Ser 2 / Ser 5** |
| **Sero-type**  **2** | 31 | Glyceraldehyde 3 phosphate dehydrogenase | GapA | B3H0Z9 | 35691 | 7.0 |  | **3.8** |
|  | 32 | Putative uncharacterized protein | APP7_1946 | B3GZB2 | 40840 | 6.5 | **3.9** | **10.8** |
|  | 33 | 5'-nucleotidase/2',3'-cyclic phosphodiesterase and related esterases | UshA | B3GXM3 | 60857 | 7.1 |  | **13.9** |
|  | 34 | Glyceraldehyde 3 phosphate dehydrogenase | GapA | B3H0Z9 | 35691 | 7.0 |  | **2.8** |
|  | 35 | Putative uncharacterized protein | APP7_1946 | B3GZB2 | 40840 | 6.5 | **3.2** | **6.1** |
|  | 36 | **Outer membrane protein P5** | OmpA1 | B3H2D9 | 39588 | 9.3 | **9.3** |  |
|  | 37 | **5'-nucleotidase/2',3'-cyclic phosphodiesterase and related esterases** | UshA | B3GXM3 | 60857 | 7.1 |  | **8.0** |
|  | 38 | Hemoglobin binding protein A | HgbA | B3H1S8 | 107545 | 9.0 |  | **9.5** |
|  | 39 | **Outer membrane protein P5** | OmpA1 | B3H2D9 | 39588 | 9.3 | **6.2** |  |
|  | 40 | **Peptidyl prolyl cis trans isomerase** | FkpA | B3H2N7 | 26479 | 7.3 |  | **10.0** |
|  | 41 | Trimethylamine N oxide reductase | TorZ | B3H1B3 | 91202 | 7.3 | **4.9** | **12.9** |
|  | 42 | RTX-IV toxin determinant A | ApxIVA | B3H1M8 | 149447 | 4.5 | **2.1** | **2.8** |
|  | 43 | **High affinity zinc uptake system protein** | ZnuA | B3H2B9 | 35710 | 6.5 |  |  |
|  | 44 | **RTX-III toxin determinant A** | ApxIIIa | P55130 | 112423 | 5.8 | **29.6** | **69.5** |
|  | 45 | **RTX-III toxin determinant A** | ApxIIIa | P55130 | 112423 | 5.8 | **28.2** | **81.7** |
|  | 46 | **RTX-III toxin determinant A** | ApxIIIa | P55130 | 112423 | 5.8 | **13.0** | **58.5** |
|  | 47 | RTX-III toxin determinant A | ApxIIIa | P55130 | 112423 | 5.8 |  | **11.4** |
|  | 48 | Phosphoglycerate kinase | Pgk | B3H222 | 46000 | 5.0 | **2.7** |  |
|  | 49 | **Outer membrane protein P5** | OmpA1 | B3H2D9 | 39588 | 9.3 | **8.9** |  |
|  | 50 | Outer membrane protein P2 | OmpP2 | B3H172 | 39571 | 9.0 | **2.4** |  |
|  | 51 | Hybrid peroxiredoxin HyPrx5 | APP7_1490 | B3H2B7 | 26924 | 5.4 | **2.8** |  |
|  | 52 | 2,3-bisphosphoglycerate-dependent phosphoglycerate mutase | GpmA | B3H1G9 | 25946 | 6.0 |  | **2.2** |
|  | 53 | Formate acetyltransferase | PflB | B3H1R7 | 86575 | 5.7 | **2.3** | **5.8** |
|  | 54 | **5'-nucleotidase/2',3'-cyclic phosphodiesterase and related esterases** | UshA | B3GXM3 | 60857 | 7.1 |  | **10.4** |
|  | 55 | **Elongation factor Tu** | TufB | B3GYJ3 | 43553 | 5.3 |  |  |
|  | 56 | **Elongation factor Tu** | TufB | B3GYJ3 | 43553 | 5.3 |  |  |
|  |  |  |  |  |  |  |  | |
|  |  |  |  |  |  |  | **Ser 5 / Ser 1** | **Ser 5 / Ser 2** |
| **Sero-type**  **5** | 57 | **Outer membrane protein P5** | OmpA1 | B3H2D9 | 39588 | 9.3 | **11.0** |  |
|  | 58 | **Outer membrane protein P5** | OmpA1 | B3H2D9 | 39588 | 9.3 | **7.1** |  |
|  | 59 | Biotin carboxylase subunit of acetyl CoA carboxylase | AccC | B3GZC1 | 49214 | 6.6 | **2.1** |  |
|  | 60 | Transferrin binding protein | TbpB | B3GYQ1 | 65489 | 7.0 | **9.2** | **5.4** |
|  | 61 | Fe3 ABC transporter iron binding protein | AfuA_2 | B3GXA4 | 37737 | 8.2 | **6.8** | **6.2** |
|  | 62 | Iron regulated outer membrane protein | Irp | B3GXR5 | 89744 | 8.6 |  | **2.2** |
|  | 63 | Outer membrane protein P2 | OmpP2 | B3H172 | 39497 | 9.1 | **11.6** |  |
|  | 64 | **Outer membrane protein P5** | OmpA1 | B3H2D9 | 39588 | 9.3 | **4.5** |  |
|  | 65 | Iron regulated outer membrane protein | Irp | B3GXR5 | 89744 | 8.6 |  | **5.0** |
|  | 66 | Predicted TonB dependent Ligand Gated channel | APP7_1350 | B3H272 | 77203 | 8.5 |  | **2.7** |
|  | 67 | 5'-nucleotidase/2',3'-cyclic phosphodiesterase and related esterases | UshA | B3GXM3 | 60857 | 7.1 | **3.4** | **2.1** |
|  | 68 | **Peptidyl prolyl cis trans isomerase** | FkpA | B3H2N7 | 26479 | 7.3 | **2.3** |  |
|  | 69 | **Transaldolase** | Tal | B3GZQ2 | 34802 | 5.1 | **2.2** |  |
|  | 70 | RTX-IV toxin determinant A | ApxIVA | B3H1M8 | 149447 | 4.5 |  | **2.8** |
|  | 71 | **ABC transport system periplasmic protein** | APP7_0756 | B3GXE9 | 32490 | 8.5 |  |  |
|  | 72 | **ABC transport system periplasmic protein** | APP7_0756 | B3GXE9 | 32490 | 8.5 |  |  |
|  | 73 | Outer membrane protein P2 | OmpP2 | B3H172 | 39571 | 9.0 | **4.4** |  |
|  | 74 | **Outer membrane protein P5** | OmpA1 | B3H2D9 | 39588 | 9.3 | **8.0** |  |
|  | 75 | Outer membrane protein P2 | OmpP2 | B3H172 | 39571 | 9.0 | **4.8** |  |
|  | 76 | RTX I toxin protein determinant A | ApxIA | Q548V0 | 109967 | 5.5 |  | **20.5** |
|  | 77 | RTX I toxin protein determinant A | ApxIA | Q548V0 | 109967 | 5.5 |  | **55.1** |
|  | 78 | **RTX I toxin protein determinant A** | ApxIA | Q548V0 | 109967 | 5.5 | **2.5** | **4.8** |
|  | 79 | **RTX I toxin protein determinant A** | ApxIA | Q548V0 | 109967 | 5.5 | **3.0** | **22.2** |
|  | 80 | RTX I toxin protein determinant A | ApxIA | Q548V0 | 109967 | 5.5 | **2.8** | **9.2** |
|  | 81 | Transferrin binding protein 1 Tbp1 | TbpA | B3GYQ0 | 106999 | 9.2 |  | **3.0** |
|  | 82 | **Iron Chelated ABC transporter periplasmic binding protein** | YfeA | B3H0B4 | 32893 | 7.5 |  | **2.6** |
|  | 83 | **30S ribosomal protein S1** | RpsA | B3GXH6 | 60227 | 5.0 | **3.8** |  |
|  | 84 | **Outer membrane protein P5** | OmpA1 | B3H2D9 | 39588 | 9.3 | **12.8** |  |
|  | 85 | Outer membrane protein P2 | OmpP2 | B3H172 | 39571 | 9.0 | **5.1** | **2.3** |
|  | 86 | Putative outer membrane protein | OmpP1 | B3GXS8 | 52907 | 9.2 | **4.4** |  |
|  | 87 | 5'-nucleotidase/2',3'-cyclic phosphodiesterase and related esterases | UshA | B3GXM3 | 60857 | 7.1 | **19.7** | **12.8** |
|  | 88 | **Protective surface antigen D15** | D15 | B3H0S5 | 89132 | 6.6 | **5.8** |  |
|  | 89 | RTX I toxin protein determinant A | ApxIA | Q548V0 | 109967 | 5.5 |  | **2.9** |
|  | 90 | ABC type Fe3 transport system periplasmic component | AfuA | B3GYH9 | 37793 | 8.4 |  | **5.2** |
|  | 91 | D galactose binding periplasmic protein | MglB2 | B3H2E0 | 35418 | 5.7 | **3.8** | **7.5** |
|  | 92 | Phosphoenolpyruvate carboxykinase ATP | PckA | B3H1D9 | 59389 | 5.6 |  | **2.2** |
|  | 93 | Putative outer membrane protein | OmpP1 | B3GXS8 | 52907 | 9.2 | **2.5** |  |
|  | 94 | Elongation factor Ts | Tsf | B3GXB1 | 30337 | 5.2 | **2.4** |  |
|  | 95 | **Elongation factor G** | FusA | B3H2G7 | 77515 | 5.1 |  |  |
|  |  |  |  |  |  |  |  |  |
|  |  |  |  |  |  |  | **Ser 7/ Pool** |  |
| **Sero-type**  **7** | 96 | Phosphoenolpyruvate carboxykinase ATP | PckA | B3H1D9 | 59389 | 5.6 | **8.7** |  |
|  | 97 | RTX II toxin determinant A | ApxIIA | B3GXU5 | 102471 | 5.6 | **3.9** |  |
|  | 98 | RTX II toxin determinant A | ApxIIA | B3GXU5 | 102471 | 5.6 | **4.7** |  |
|  | 99 | Phosphoglucomutase phosphomannomutase | Pgm | B3H119 | 59532 | 5.3 | **2.3** |  |
|  | 100 | RTX II toxin determinant A | ApxIIA | B3GXU5 | 102471 | 5.6 | **7.5** |  |
|  | 101 | Hemoglobin binding protein A | HgbA | B3H1S8 | 107545 | 9.0 | **13.6** |  |
|  | 102 | RTX II toxin determinant A | ApxIIA | B3GXU5 | 102471 | 5.6 | **5.8** |  |
|  | 103 | **Protective surface antigen D15** | D15 | B3H0S5 | 89132 | 6.6 |  |  |
|  | 104 | Phosphoenolpyruvate carboxykinase ATP | PckA | B3H1D9 | 59389 | 5.6 | **4.9** |  |
|  | 105 | RTX II toxin determinant A | ApxIIA | B3GXU5 | 102471 | 5.6 | **3.4** |  |
|  | 106 | RTX II toxin determinant A | ApxIIA | B3GXU5 | 102471 | 5.6 | **5.3** |  |
|  | 107 | **5'-nucleotidase/2',3'-cyclic phosphodiesterase and related esterases** | UshA | B3GXM3 | 60857 | 7.1 | **4.7** |  |
|  | 108 | Putative outer membrane protein | OmpP1 | B3GXS8 | 52907 | 9.2 | **2.6** |  |
|  | 109 | ABC type Fe3 transport system periplasmic component | AfuA | B3GYH9 | 37793 | 8.4 | **12.3** |  |
|  | 110 | RTX II toxin determinant A | ApxIIA | B3GXU5 | 102471 | 5.6 | **6.2** |  |
|  | 111 | Phosphoglucomutase phosphomannomutase | Pgm | B3H119 | 59532 | 5.3 | **2.2** |  |
|  | 112 | **Protective surface antigen D15** | D15 | B3H0S5 | 89132 | 6.6 |  |  |
|  | 113 | Iron Chelated ABC transporter periplasmic binding protein | YfeA | B3H0B4 | 32893 | 7.5 | **3.5** |  |
|  | 114 | **RTX II toxin determinant A** | ApxIIA | B3GXU5 | 102471 | 5.6 |  |  |
|  | 115 | FKBP-type peptidyl-prolyl cis-trans isomerases 1 | FkpA | B3H2N7 | 26479 | 7.3 | **3.7** |  |
|  | 116 | **5'-nucleotidase/2',3'-cyclic phosphodiesterase and related esterases** | UshA | B3GXM3 | 60857 | 7.1 | **3.3** |  |
|  | 117 | Hemoglobin binding protein A | HgbA | B3H1S8 | 107545 | 9.0 | **13.6** |  |
|  | 118 | **Outer membrane protein P5** | OmpA1 | B3H2D9 | 39588 | 9.3 | **3.8** |  |
|  | 119 | **Outer membrane protein P5** | OmpA1 | B3H2D9 | 39588 | 9.3 | **6.0** |  |
|  | 120 | **GTPases - translation elongation factors** | TufB | B3GYJ3 | 43553 | 5.3 |  |  |
|  | 121 | **GTPases - translation elongation factors** | TufB | B3GYJ3 | 43553 | 5.3 |  |  |
|  | 122 | **Outer membrane protein P5 (OMP P5)** | OmpA2 | B3GZA8 | 38806 | 9.5 |  |  |
|  | 123 | **protective surface antigen D15** | D15 | B3H0S5 | 89132 | 6.6 |  |  |
|  | 124 | **Iron-regulated outer membrane protein B** | FrpB | B3H0B8 | 73081 | 9.6 |  |  |
|  | 125 | **RTX-II toxin determinant A** | ApxIIA | B3GXU5 | 102471 | 5.6 |  |  |
|  | 126 | **RTX-II toxin determinant A** | ApxIIA | B3GXU5 | 102471 | 5.6 | **4.7** |  |
|  | 127 | **RTX-II toxin determinant A** | ApxIIA | B3GXU5 | 102471 | 5.6 | **7.5** |  |
|  | 128 | **RTX-II toxin determinant A** | ApxIIA | B3GXU5 | 102471 | 5.6 | **9.4** |  |
|  | 129 | **RTX-II toxin determinant A** | ApxIIA | B3GXU5 | 102471 | 5.6 | **12.0** |  |
|  | 130 | **RTX-II toxin determinant A** | ApxIIA | B3GXU5 | 102471 | 5.6 | **19.1** |  |
|  | 131 | iron (chelated) ABC transporter, periplasmic- binding protein | YfeA | B3H0B4 | 32893 | 6.8 | **5.1** |  |
|  | 132 | phosphoglucomutase/phosphomannomutase | Pgm | B3H119 | 59532 | 5.3 | **2.2** |  |
|  | 133 | **Outer membrane protein P5** | OmpA1 | B3H2D9 | 39588 | 9.3 | **4.9** |  |
|  | 134 | RTX-II toxin determinant A | ApxIIA | B3GXU5 | 102471 | 5.6 | **3.7** |  |
|  | 135 | RTX-II toxin determinant A | ApxIIA | B3GXU5 | 102471 | 5.6 | **21.5** |  |
|  | 136 | RTX-II toxin determinant A | ApxIIA | B3GXU5 | 102471 | 5.6 | **14.8** |  |
|  | 137 | RTX-II toxin determinant A | ApxIIA | B3GXU5 | 102471 | 5.6 | **15.3** |  |

Protein identifications from preparative 2-D gels of the different serotypes. Proteins selected for mass spectrometric analysis either showed relative spot intensities above a threshold of two as obtained by 2-D DIGE analyses (Figure 1) or were cross-serotype reactive as obtained by 2-D immunoblotting (Figure 2).

^a)^ In order to have a consistent nomenclature for proteins identified from *A. pleuropneumoniae* serotypes 1, 2, 5 or 7 in the columns “protein description”, “protein” and “accession number”, the annotation of the respective homologue of *A. pleuropneumoniae* serotype 7 was adopted from the uniprot knowledgebase (http://www.uniprot.org/). For the Apx toxins ApxIA and ApxIIIA, which are not present in *A. pleuropneumoniae* serotype 7, the common nomenclature was used.

^b)^ Immunogenic proteins (Figure 2) are highlighted in bold.
