# Supplementary material for: Proteomic and immunoproteomic characterization of a DIVA subunit vaccine against Actinobacillus pleuropneumoniae
Source: Proteome Sci. 2011 Apr 20;9:23. doi: 10.1186/1477-5956-9-23 (PMC3107771; doi:10.1186/1477-5956-9-23)
Supplement: Additional file 4 — Figure S2: Preparative 2-D gels of "detergent-wash" proteins. "Detergent-wash" proteins of serotypes 1, 2, 5 and 7 were separated on individual 2-D gels. Proteins were stained with colloidal Coomassie blue and proteins of interest were picked from these gels, trypsinized and analyzed by mass spectrometry. The numbers on each spot, that has been identified, are consecutive and allow the finding of the respective spot in Figure 1 and 2. [file 1477-5956-9-23-S4.PPT]

## Slide 1
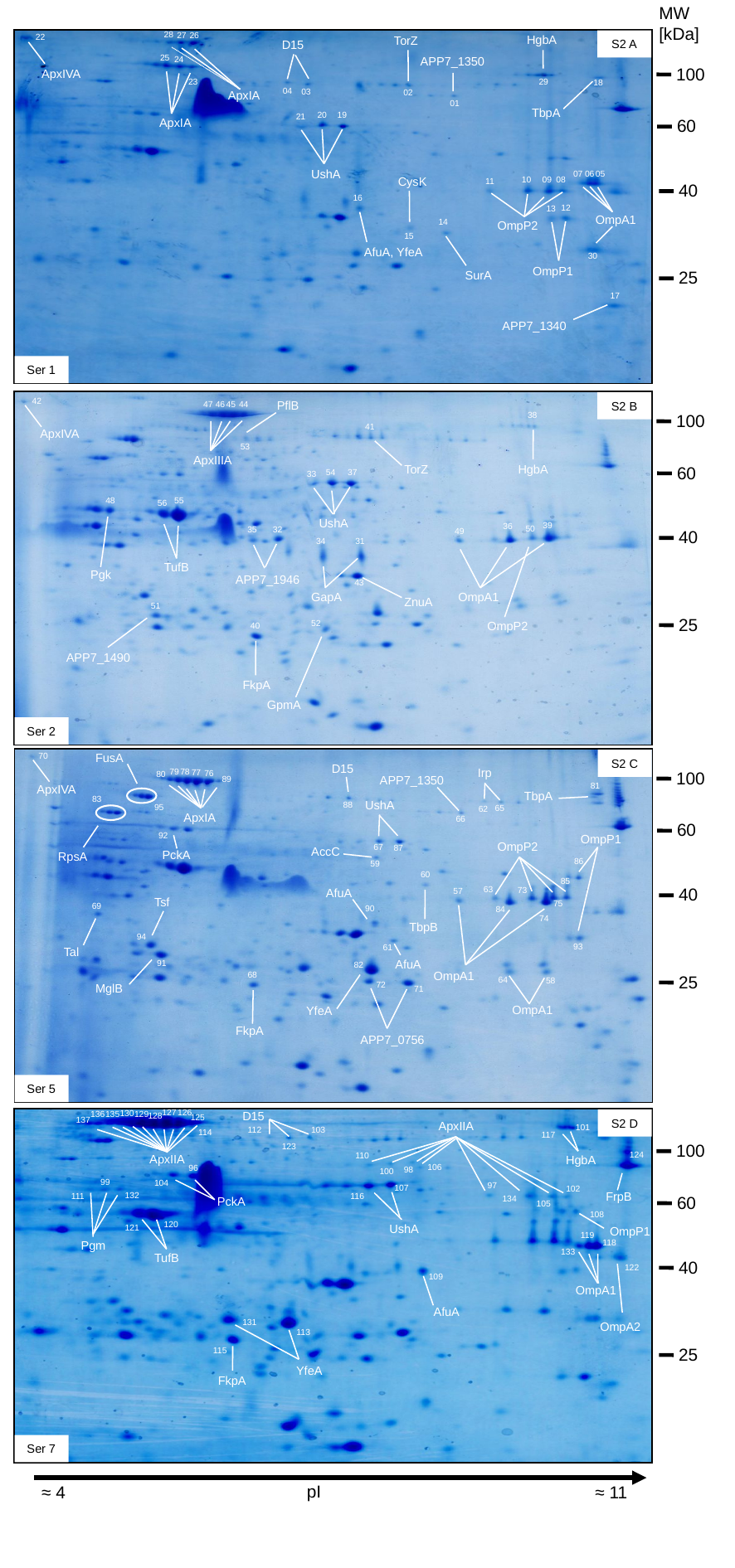

MW [kDa]
28
27
26
HgbA
22
TorZ
S2 A
D15
25
APP7_1350
24
100
ApxIVA
23
29
18
04
03
ApxIA
02
01
TbpA
20
19
21
ApxIA
60
UshA
07
06
05
CysK
10
09
08
11
40
16
12
13
OmpA1
14
OmpP2
15
AfuA, YfeA
30
OmpP1
25
SurA
17
APP7_1340
Ser 1
42
PflB
S2 B
46
45
47
44
100
38
41
ApxIVA
53
ApxIIIA
HgbA
TorZ
60
54
37
33
55
48
56
UshA
39
36
50
32
35
40
49
34
31
TufB
Pgk
APP7_1946
43
GapA
OmpA1
ZnuA
51
25
OmpP2
52
40
APP7_1490
FkpA
GpmA
Ser 2
FusA
70
S2 C
D15
Irp
78
100
79
77
76
80
APP7_1350
89
ApxIVA
81
TbpA
83
UshA
88
95
65
62
ApxIA
66
60
92
OmpP1
OmpP2
67
87
AccC
PckA
RpsA
86
59
60
85
40
AfuA
63
73
57
Tsf
75
69
90
84
74
TbpB
94
93
61
Tal
AfuA
91
82
OmpA1
68
25
64
58
72
MglB
71
OmpA1
YfeA
FkpA
APP7_0756
Ser 5
126
127
D15
130
136
135
129
128
125
137
S2 D
ApxIIA
101
112
103
114
117
100
123
124
ApxIIA
110
HgbA
106
96
98
100
99
104
97
107
102
FrpB
132
111
116
60
PckA
134
105
108
120
UshA
121
OmpP1
119
Pgm
118
133
TufB
40
122
109
OmpA1
AfuA
131
OmpA2
113
25
115
YfeA
FkpA
Ser 7
pI
≈ 4
≈ 11
